# Free Energy Surfaces and Barriers for Vacancy Diffusion on Al(100), Al(110), Al(111) Reconstructed Surfaces

**DOI:** 10.3390/nano12010076

**Published:** 2021-12-28

**Authors:** Junais Habeeb Mokkath, Mufasila Mumthaz Muhammed, Ali J. Chamkha

**Affiliations:** 1 Quantum Nanophotonics Simulations Lab, Department of Physics, Kuwait College of Science and Technology, Doha Area, 7th Ring Road, Kuwait City P.O. Box 27235, Kuwait; 2 School of Engineering & Computing, American International University, Saad Al Abdullah-East of Naseem, Block 3, Kuwait; m.muhammed@aiu.edu.kw; 3 Faculty of Engineering, Kuwait College of Science and Technology, Doha 35004, Kuwait; a.chamkha@kcst.edu.kw

**Keywords:** metadynamics, diffusion

## Abstract

Metadynamics is a popular enhanced sampling method based on the recurrent application of a history-dependent adaptive bias potential that is a function of a selected number of appropriately chosen collective variables. In this work, using metadynamics simulations, we performed a computational study for the diffusion of vacancies on three different Al surfaces [reconstructed Al(100), Al(110), and Al(111) surfaces]. We explored the free energy landscape of diffusion and estimated the barriers associated with this process on each surface. It is found that the surfaces are unique regarding vacancy diffusion. More specically, the reconstructed Al(110) surface presents four metastable states on the free energy surface having sizable and connected passage-ways with an energy barrier of height 0.55 eV. On the other hand, the reconstructed Al(100)/Al(111) surfaces exhibit two/three metastable states, respectively, with an energy barrier of height 0.33 eV. The findings in this study can help to understand surface vacancy diffusion in technologically relevant Al surfaces.

## 1. Introduction

Molecular dynamics (MD) simulations have been shown to be an indispensable toolkit for efficient exploration of the configurational space of complex systems, since they render dynamical evolution with in-depth atomistic level details. Notwithstanding recent advancements in purpose-built computational resources and software tools, the timescales of milliseconds and beyond remains a longstanding (inherent) problem for MD simulations [1,2]. Consequentially, the possibility of understanding longtime scale phenomena like “rare events” (more specifically, an event happening at low frequency such as protein folding and nucleation) is still beyond the reach of MD simulations. To overcome this limitation, significant effort has been devoted to developing a great variety of enhanced sampling methods [3,4,5,6,7,8,9,10,11,12,13,14,15,16,17]. Metadynamics (MetaD) [18,19,20,21,22,23,24,25,26,27,28] is one such well-established enhanced sampling method which has been successfully applied to a variety of areas in biophysics and material science, to list a few. The power of MetaD simulation is its simplicity: it captures rare events and maps out the underlying free energy surface. MetaD utilizes the recurrent application of a history dependent adaptive bias potential (that helps to locate regions in the configurational space separated by high energy barriers and enhance minima-to-minima transitions) to a limited number of degrees of freedom called collective variables (CVs) [29,30,31,32,33]. Over the last few years, a multitude of CVs have been employed including coordination numbers, lattice parameters, interatomic angles, and potential energy, to mention a few. More specifically, CVs provide a low-dimensional projection of the configurational space and are selected to describe modes of the system that are more difficult to explore. Also, note that the different metastable states of a system should correspond to different values of the CVs.

In this work, we employ MetaD simulations to study the free energy surfaces and free energy barriers of low-index Al(100), Al(110), and Al(111) systems having a surface vacancy. Al is of particular interest recently due to its exceptional properties [34,35,36,37,38,39]. On the other hand, defects like vacancy are omnipresent on crystals and play an important role in their properties [40,41,42,43,44]. Substantial experimental and theoretical efforts have been employed over many years to elucidate the free energy surfaces and free energy barriers of diffusion on crystal surfaces [45,46,47,48]. How diffusion happens on the atomic scale on a crystal surface is a fundamental problem, and a detailed knowledge of the diffusion process is crucial for applications that demand high technological precision [49,50,51]. Importantly, vacancy diffusion is a thermally activated process demanding the system to overcome free energy barriers. Generally, free energy barriers are higher than thermal energy, and therefore, the diffusion process is regarded as a rare event.

A detailed understanding of free energy surfaces and free energy barriers are essential cornerstones for the design and fabrication of nanodevices with enhanced performance. While experiments face challenges in identifying and characterizing diffusion processes, simulation approaches can predict them accurately. To our knowledge, vacancy diffusion on reconstructed Al surfaces has not been investigated yet by MetaD simulations. Our paper aims at filling this gap. The remainder of this paper is organized as follows. In the following section, we provide the details of the computational methodology we used. Next, we discuss the important results from our simulations, such as the radial distribution functions, mass density profiles, two-dimensional free energy surfaces, and free energy barriers on the reconstructed Al(100), Al(110), and Al(111) surfaces. As a key result in this study, the highest barrier for vacancy diffusion is found in the case of Al(110) surface due to its more open nature. The results obtained in this work would help in a better understanding of the modulations in the vacancy diffusion process on different low-index Al surfaces, beneficial for Al based technology.

## 2. System and Computational Aspects

**Figure 1:**
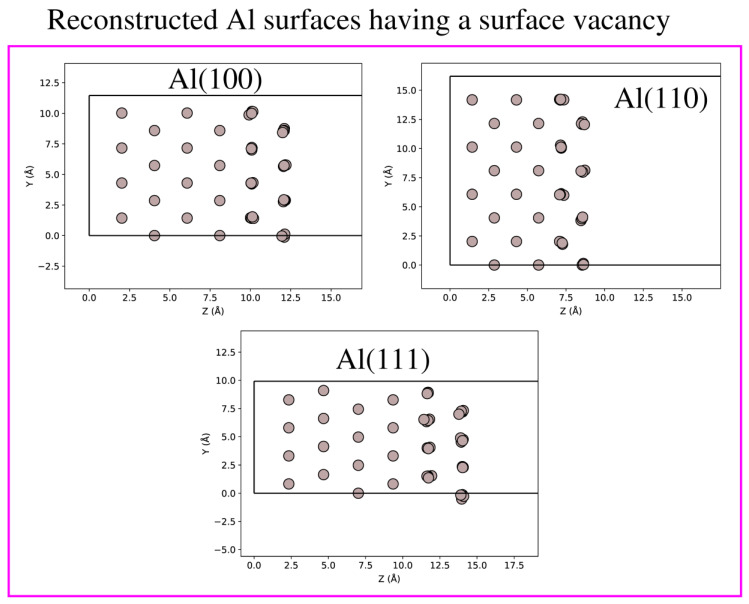
Reconstructed Al(100), Al(110), Al(111) slabs having a surface vacancy. Note that the atoms in the two top most layers were allowed to relax while the atoms occupying the four bottom layers were fixed.

MetaD simulations were carried out using a standard protocol by the PLUMED software version 2.1 [52] interfaced to the QuantumWise Atomistix ToolKit (QuantumATK) software package [53]. This plugin provides customized software libraries for a variety of CVs and also renders several post-processing tools. Our MetaD simulations involve several steps: (a) modeling of Al(100), Al(110), Al(111) surfaces and definition of Al-Al atomic interaction; (b) governing equations for the system, conditions of simulation and energy minimization; and (c) integration scheme and calculation of concerned properties. Now, regarding the systems under investigation, we created low-index Al(100), Al(110), Al(111) slabs with 4 × 4 surface lattice from the bulk Al. Each of these slabs is composed of six layers with a total number of 96 atoms. Nevertheless, the slab thickness is different from each other. The Al-Al atomic interactions were described using the EAM (embedded atom method) potential [54]. Briefly, the EAM potential can be written as: $E_{\mathrm{tot}}=\frac{1}{2}\underset{i\neq j}{\sum }V\left(r_{ij}\right)+\underset{i}{\sum }F\left(\rho_{i}\right)$ with $\rho_{i}=\underset{j}{\sum }\phi \left(r_{ij}\right)$ where *E*_tot_ is the total energy, $V\left(r_{ij}\right)$ the pair potential, $F\left(\rho_{i}\right)$ the embedding function, and $\phi \left(r_{ij}\right)$ the electron density contribution from atom *j* to atom *i*. The total electron density *ρ_i_* at an atom position *i* is calculated via the linear superposition of electron density contributions from neighboring atoms. For each MetaD simulation run, a single Al surface vacancy was generated by removing the atom of the topmost layer with position *x* = 0 and *y* = 0. In the next step, we performed structure optimization. In all slabs, we allowed the atoms occupying two top most layers to relax, while the atomic positions of those atoms occupying the four bottom layers were kept fixed to mimic bulk constraints; see Figure 1. To avoid the spurious interaction between the slab and its periodic images, a vacuum distance of 15 Å used along the perpendicular direction to the slab.

Each simulation run was force-minimized and then equilibrated for 10,000,000 timesteps using Langevin method [55,56]. Since the starting Al(100), Al(110), and Al(111) slab structures will relax because of the vacancy creation, MetaD was started only after 10,000 time steps, which was sufficient for relaxation. We have chosen two CVs defined by distance traveled by the atom *p* (an atom on the surface just next to the vacancy) along the Cartesian axes, *x* and *y* (with no bias in the direction or pathway of migration). Minima-to-minima transitions were made possible within the simulation timescale via a history-dependent bias potential [52] (applied to all atoms in the two top most layers) constructed by summing Gaussian contributions of height *h* and width *w* in the space defined by the CV as follows:

(1) $$\Delta V\left(\eta \right)=\underset{k<nG}{\sum }w_{k}\;\exp\left[\frac{\left(\eta -\eta \left(k\tau G\right)\right)}{2\delta^{2}}\right]$$

where ∆V(*η*) is the history-dependent bias potential, *η* is the CV used, *τ* is the Gaussian deposition stride, *w* and *δ* are the Gaussian height and width, respectively. The ∆V(*η*) is updated, and while the simulation runs, it fills the underlying free energy surface helping the crossing of the energy barriers. We remark that several test runs were first performed to optimize the various simulation parameters such as the height and width of the Gaussians. We choose *w* = 0.05 eV and *δ* = 0.025 eV considering the accuracy and CPU time. Gaussians were deposited every 1000 time-steps. It should be stressed that ∆V(*_η_*) bias encourages the sampling of the unfavorable states by kicking the system out of the most favorable ones.

## 3. Results and Discussion

**Figure 2:**
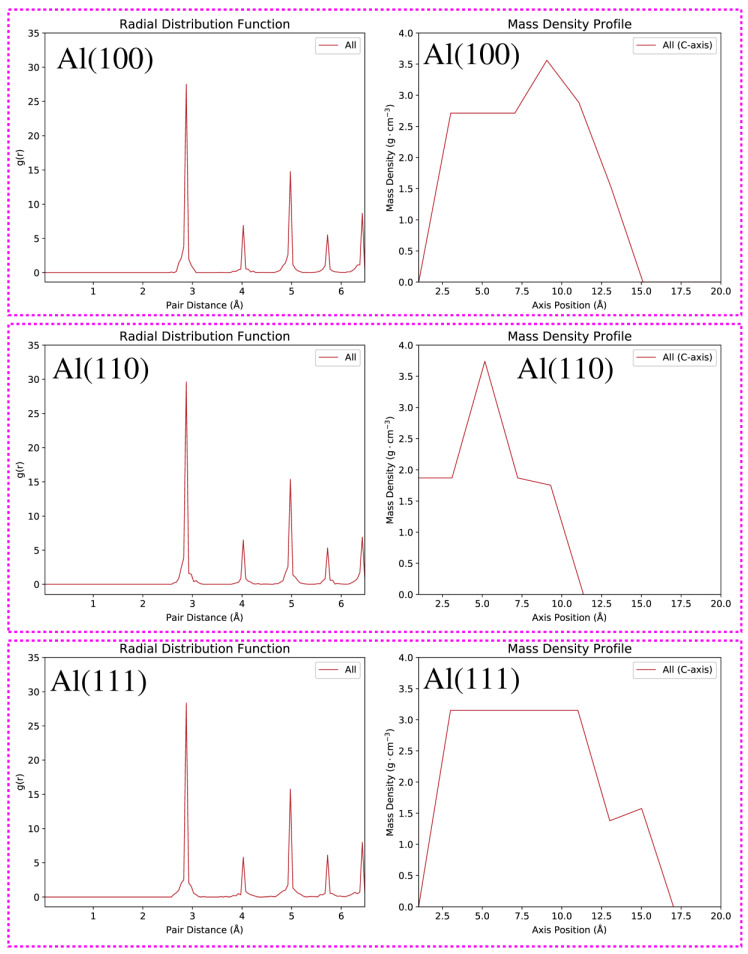
RDFs (**left** panel) and mass density profiles (**right** panel) of the reconstructed Al(100), Al(110), Al(111) slabs having a surface vacancy.

Before we begin our analysis and discussion on the free energy surfaces and free energy barriers of low-index Al(100), Al(110), Al(111) surfaces, it is informative to provide first the results of the structure optimizations, see Figure 1. In this context, we provide the radial distribution functions (RDFs) and mass density profiles of the reconstructed Al(100), Al(110), Al(111) surfaces, see Figure 2. We have calculated the RDFs (see the left panel of Figure 2) using the equation $g\left(r\right)=\frac{1}{4\pi r^{2}}\frac{1}{N\rho }\overset{N}{\underset{i=1}{\sum }}\overset{N}{\underset{j\neq i}{\sum }}\langle \delta \left(r-\left|r_{i}-r_{j}\right|\right)\rangle$. As common to all the investigated systems, the RDFs show a pronounced peak at around 2.875 Å, indicating that this is the dominant nearest-neighbor distance between Al atoms. This value is very close to the nearest-neighbor distance between Al atoms in crystalline Al (2.856 Å) at room temperature [57]. Next, we analyse the mass density profiles, see the right panel of Figure 2. It shows how mass is distributed along the perpendicular direction to the slab. One easily concludes that mass density profiles are markedly different from each other due to the unique distributions of Al atoms in the Al(100), Al(110), and Al(111) configurations. Additionally, small modulations to the mass density profiles are also contributed by atom displacements during structure optimization. It is noteworthy that Al(100), Al(110), and Al(111) mass density profiles plummeted to zero at around 15.10 Å, 11.50 Å, and 17.20 Å, respectively. This result is in agreement with the fact that Al(110) slab is more compact and open in comparison to the Al(100) and Al(111) slabs, see Figure 1. It is also important to emphasize that an inward relaxation of the top layer is observed in all reconstructed structures, see Figure 1. This arises due to the surface asymmetry, where the force of attraction will be greater for bulk atoms causing a significant inward relaxation of the top surface layer [58].

**Figure 3:**
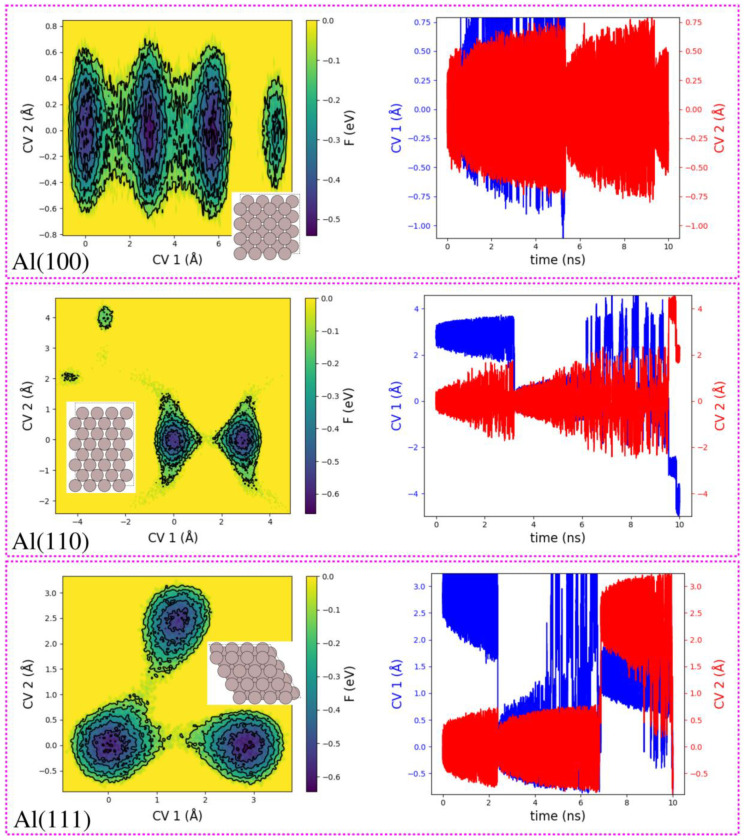
Plots showing the free energy surfaces as a function of the chosen CVs (**left** panel) and evolution of the CVs over the simulation time in nanoseconds (**right** panel) of the reconstructed Al(100), Al(110), and Al(111) slabs having a surface atomic vacancy. All energy values are in eV and all distance values are in Å.

After having analysed and discussed the structural features of the reconstructed Al(100), Al(110), Al(111) slabs having a surface vacancy, next we investigate the free energy surfaces [F(s)] as a function of the CV1 and CV2, see the left panel in Figure 3. The plots show a heat map of F(s) as a function of the chosen CVs. It is noteworthy that F(s) corresponding to Al(100), Al(110) and Al(111) structures yield unique and interesting features as being composed of different metastable states. More specifically, in the case of Al(100) slab, one finds four basins or metastable states on the free energy surface (see the top left panel in Figure 3), corresponding to the diffusion of the vacancy from one site to the neighboring one. Interestingly, one observes that the three metastable states are well connected by sizable passageways. It is important to mention that metastable states that are connected by passageways allow rare but crucial transitions from one state to another. Additionally, it is noteworthy that with chosen CV1 and CV2, the F(s) clearly separate the metastable states. In the case of Al(100) structure, one finds that CV1 can distinguish the four different metastable states, while CV2 is not. Technically speaking, metastability originates when the probability distribution as a function of the atomic coordinates has at least two peaks separated by a region in which the probability is many orders of magnitude lower. The metastable states displayed in Figure 3 are akin to an archetypal example in which a molecule that can undergo a chemical reaction [59]. In the case of A(110) and Al(111) slabs, one finds two and three metastable states on the free energy surface, respectively. More specifically, in the case of Al(110) slab, the two metastable states are connected by a narrow passageway and CV1 is crucial than CV2 since the former clearly separates the two available metastable states. The free energy surface of Al(111) is more interesting. One finds that metastable states are positioned more or less on the corners of a triangle with no passageway on one side. On the other hand, CV1 again appears to be the best performing CV, nonetheless, CV2 can also be helpful in identifying at least two metastable states on the free energy surface.

It is also interesting to analyse how the collective variables, CV1 and CV2 evolve over simulation time, see the right panel of Figure 3. More specifically, in the case of Al(100) slab, one finds that CV2, which corresponds to the *y* Cartesian coordinate, oscillates around a constant value of 0 Å. This is because all the three (connected) metastable states appear at the same *y* value. The first, second, and third metastable states are filled until at approximately 5.20 ns, 9.50 ns, and 10.10 ns, respectively. In contrast, the evolution of CV1, which corresponds to the *x* Cartesian coordinate, suggests that it is filled until at approximately 5.20 ns. Now let us move onto Al(110) slab. One finds that CV2, which corresponds to the *y* Cartesian coordinate, oscillates around a constant value of 0 Å, because both the metastable states occur at the same *y* position. One finds that both CV1 and CV2 are filled at approximately 3.20 ns. Finally, in the case of Al(111), CV2 is filled at approximately 2.30 ns and 6.80 ns. However, in the case of CV1, it is filled at approximately 2.30 ns. Then it goes to the second metastable state oscillating around 0 Å and jump to the third metastable state oscillating around 1.50 Å.

**Figure 4:**
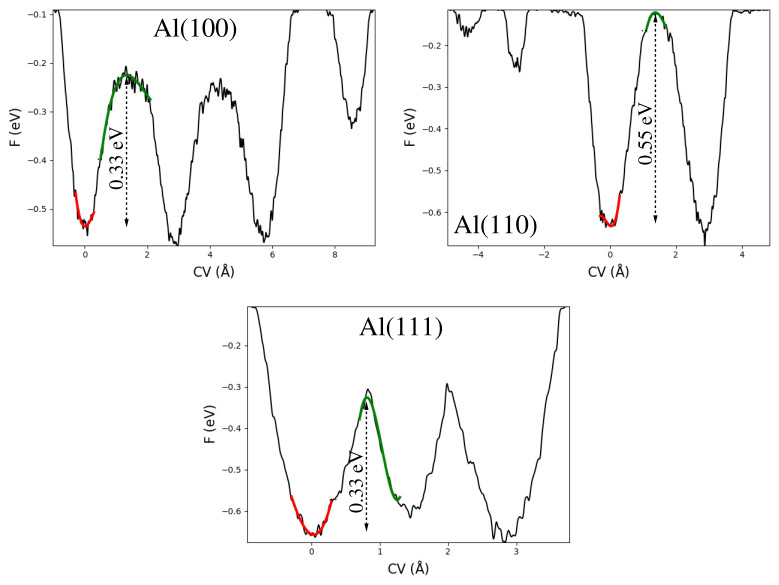
The free energy barriers on the reconstructed Al(100), Al(110), and Al(111) slabs having a surface vacancy. All energy values are in eV and all distance values are in Å.

Having analysed and discussed the free energy surfaces and evolution of CVs over simulation time in detail, it is worthwhile to estimate the free energy barrier for surface vacancy diffusion at low-index Al(100), Al(110), Al(111) surfaces, see Figure 4. One finds that the free energy barrier of Al(100) and Al(111) slabs amount to 0.33 eV (thus energetically very similar), while the free energy barrier of Al(110) amounts to a higher value of 0.55 eV. These results are in relatively good agreement with the previous work reporting vacancy diffusion on the different Al surfaces [28]. Vacancy diffusion at the Al(110) surface has twice the barrier as Al(100) and Al(111) surfaces is highly likely due to the open nature of the Al(110) surface. As expected, on an open surface, vacancy diffusion finds it more difficult since it has to overcome large energy barriers. These results are in tune with the previous MetaD study (Cu surfaces) finding that there are strong differences in the mobilities of the vacancies depending on the specific surface [60]. Finally, it is worthwhile to mention that ref. [28] reported two energy barriers for adatom diffusion on Al(110) surface; one parallel to the surface and other perpendicular to the surface. Nevertheless, in the present study, we found a single energy barrier for vacancy diffusion on Al(110) surface. This is due to differences in the diffusion process.

## 4. Summary and Outlook

Exploring and quantifying free energy surfaces (FESs) is the key to the comprehension of a plethora of phenomena in chemistry, materials science, and biophysics. Generally, FESs are characterized by several metastable states separated by large free energy barriers. To overcome these barriers and efficiently sample configurational space, it is worthwhile to employ enhanced sampling techniques such as MetaD simulations. Here, we have presented a detailed study elucidating the free energy surfaces and free energy barriers for surface vacancy diffusion on low-index Al surfaces using MetaD simulations. With the use of these exemplary reconstructed surfaces, we reveal the unique vacancy diffusion process. The reconstructed Al(110) surface presents four metastable states on the FES. Among them, three metastable states are well connected by sizable passageways leading to rare but crucial transitions. On the other hand, the reconstructed Al(110) structure presents two metastable states that are connected by a narrow passageway and the reconstructed Al(111) surface presents three metastable states that are positioned on the three corners of a triangle. The results in this study will help to understand Al surfaces with vacancy defects which are necessary to design highly efficient Al based devices. While here we have restricted our work to Al vacancy diffusion, this work can be considered as a step toward the general structural and dynamical characterization of other types of defects.

## Data Availability

The data presented in this study are available on request from the corresponding author.

## Nomenclature

Å	Angstrom
CV	Collective variable
EAM	Embedded atom method
F(s)	Free energy surface
MetaD	Metadynamic
ns	Nanosecond
RDF	Radial distribution function
